# Cellular Immune Response and T Cell Epitope Mapping of *Plasmodium falciparum* Chimeric Vaccine Candidate GMZ2.6c and Its Components (MSP-3, GLURP and Pfs48/45) in Individuals Naturally Exposed to Malaria in Brazilian Amazon

**DOI:** 10.3390/vaccines14050423

**Published:** 2026-05-08

**Authors:** Barbara de Oliveira Baptista, Isabela Ferreira Soares, Hugo Amorim dos Santos de Souza, Jenifer Peixoto de Barros, Evelyn Kety Pratt Riccio, Rodrigo Medeiros Martorano, Rodrigo Nunes Rodrigues-da-Silva, Linda Eva Amoah, Susheel Kumar Singh, Michael Theisen, Josué da Costa Lima-Junior, Paulo Renato Rivas Totino, Cláudio Tadeu Daniel-Ribeiro, Lilian Rose Pratt-Riccio

**Affiliations:** 1 Laboratório de Pesquisa em Malária, Instituto Oswaldo Cruz, Fundação Oswaldo Cruz, Rio de Janeiro 21040-900, RJ, Brazil; barbara.baptista@ioc.fiocruz.br (B.d.O.B.); hugosouza@aluno.fiocruz.br (H.A.d.S.d.S.); jeniferbarros@aluno.fiocruz.br (J.P.d.B.); ericciovazoler@hotmail.com (E.K.P.R.); prtotino@ioc.fiocruz.br (P.R.R.T.); malaria@fiocruz.br (C.T.D.-R.); 2 Centro de Pesquisa, Diagnóstico e Treinamento em Malária (CPD-Mal), Fundação Oswaldo Cruz e Secretaria de Vigilância em Saúde e Ambiente, Ministério da Saúde, Rio de Janeiro 21040-900, RJ, Brazil; 3 Laboratório de Imunoparasitologia, Instituto Oswaldo Cruz, Fundação Oswaldo Cruz, Rio de Janeiro 21040-900, RJ, Brazil; isaferreirasoares@gmail.com (I.F.S.); josue@ioc.fiocruz.br (J.d.C.L.-J.); 4 Laboratório de Doenças Infecciosas na Amazônia Ocidental, Universidade Federal do Acre–Campus Floresta (UFAC), Cruzeiro do Sul 69895-000, AC, Brazil; rodrigo.martorano@ufac.br; 5 Laboratório de Hantaviroses e Rickettsioses, Instituto Oswaldo Cruz, Fundação Oswaldo Cruz, Rio de Janeiro 21040-900, RJ, Brazil; nunes@ioc.fiocruz.br; 6 Immunology Department, Noguchi Memorial Institute for Medical Research, University of Ghana, Accra P.O. Box LG 25, Ghana; levaamoah@noguchi.ug.edu.gh; 7 Centre for Translational Medicine and Parasitology, Department for Immunology and Microbiology, Faculty of Health and Medical Sciences, University of Copenhagen, DK-2200 Copenhagen, Denmark; skbaghel@gmail.com (S.K.S.); mth@ssi.dk (M.T.); 8 Statens Serum Institut (SSI), DK-2300 Copenhagen, Denmark

**Keywords:** malaria vaccine, *Plasmodium falciparum*, GMZ2.6c, cellular immune response, T cell epitope mapping

## Abstract

Background/Objectives: The GMZ2.6c malaria vaccine candidate is a multi-stage *P. falciparum* chimeric protein that contains a fragment of the sexual stage Pfs48/45-6c protein genetically fused to GMZ2, which is an asexual stage vaccine construct consisting of conserved domains of Glutamate-Rich Protein (GLURP) and Merozoite Surface Protein-3 (MSP-3). Previous studies showed that GMZ2.6c is widely recognized by antibodies from individuals living in endemic areas of Brazil and that levels of anti-GMZ2.6c increase with malaria exposure and may contribute to immunity against the parasite. As cell-mediated responses are crucial for parasite control and protection, identifying antigens that elicit antigen-specific T cell recall in naturally exposed populations is the key to vaccine development. This study aimed to evaluate the cellular immune response against GMZ2.6c and its components (MSP-3, GLURP, and Pfs48/45) and to identify promiscuous T cell epitopes in individuals exposed to malaria in the Brazilian Amazon, considering the impact of active *P. falciparum* infection on antigen-specific T cell recall. Methods: This study was carried out using peripheral blood mononuclear cells (PBMCs) from individuals with active *P. falciparum* infection (PFI) and non-infected individuals exposed to malaria (NI) from Cruzeiro do Sul and Mâncio Lima, Acre State, and Guajará, Amazonas State. The PBMCs were stimulated with GMZ2.6c and its components, and cellular activation, CD4^+^ and CD8^+^ memory T cell subsets, and cytokine production were evaluated by flow cytometry. IFN-γ-secreting T cells were quantified by ELISpot using predicted T cell epitopes. Results: The individuals infected by *P. falciparum* displayed more CD8^+^ T cell activation in response to MSP-3 and Pfs48/45 and an increase in CD4^+^ T_CM_ cells and a reduction in CD4^+^ T_EM_ cells following stimulation with Pfs48/45 and GMZ2.6c. The PBMCs from both groups showed elevated production of IL-6 and TNF after stimulation with GMZ2.6c, MSP-3, and Pfs48/45, but only the non-infected individuals had high levels of IL-10. T cell epitope prediction identified sequences within MSP-3, GLURP, and Pfs48/45 that elicited IFN-γ responses in both the non-infected and *P. falciparum*-infected individuals. Conclusions: Individuals exhibit cellular immune responses to MSP-3 and Pfs48/45 that are recalled following GMZ2.6c stimulation. *P. falciparum* infection may modulate immune response, inducing a prominent pro-inflammatory response. Conversely, in the absence of the parasite, the individuals displayed balanced Th1/Th2 cytokine production. Several promiscuous T cell epitopes were able to recall IFN-γ responses. Further studies are needed to fully ascertain the potential of GMZ2.6c as a protective candidate vaccine against malaria.

## 1. Introduction

Malaria is a vector-borne tropical and subtropical infectious disease caused by apicomplexan parasites of the genus *Plasmodium*. Despite the successful efforts to reduce the global malaria burden, the disease remains a major public health challenge, causing 263 million cases and 597,000 deaths in 2023, the vast majority attributed to *P. falciparum* [1]. Current strategies for malaria control include vector control, chemoprophylaxis, and prompt diagnosis, followed by adequate treatment. However, the emergence of parasite resistance to artemisinin and its partner drugs in Artemisinin-based Combination Therapies (ACTs) [2,3,4,5,6,7,8,9], *Anopheles’* resistance to the available insecticide classes [10], genetic mutations in *P. falciparum* that impair detection using Rapid Diagnostic Tests (RDTs) [11,12,13,14], and the invasion and spread of the Asian invasive malaria vector *An. stephensi* [15] highlights the need for an effective vaccine to complement the existing tools in the elimination and eradication of malaria. Recently, the World Health Organization (WHO) approved two malaria vaccines, RTS,S/AS01 and R21/Matrix-M, recommended for children in regions with moderate to high *P. falciparum* transmission levels [16,17]. The RTS,S/AS01 vaccine showed modest and limited effectiveness, preventing approximately 30% of severe cases, 21% of hospital admissions with malaria parasitemia, and 13% of childhood deaths, while R21/Matrix-M was shown to reduce the number of symptomatic malaria cases by 75% at phase 3 clinical trials [16,17,18,19]. Nevertheless, both these malaria vaccines presented reduced vaccine efficacy over the first 12 months of follow-up. It is crucial to develop malaria vaccine candidates capable of inducing robust and long-lasting protection.

The GMZ2.6c malaria vaccine candidate is a multi-stage *P. falciparum* chimeric protein that contains a fragment of the sexual stage Pfs48/45-6c protein genetically fused to the recombinant protein GMZ2, which is an asexual stage vaccine construct consisting of conserved domains of Glutamate-Rich Protein (GLURP) and Merozoite Surface Protein-3 (MSP-3) expressed in *Lactococcus lactis* [20]. Previous studies showed that mice immunized with GMZ2.6c formulations containing the synthetic TLR4 agonist glucopyranosyl lipid adjuvant (GLA) or a synthetic lipid adjuvant (SLA) presented high and functional specific antibody titers against sexual and asexual stage antigens of *P. falciparum* and enhanced antigen-specific CD4+ Th1 cells secreting both interferon-gamma (IFN-γ) and tumor necrosis factor (TNF) in response to the GMZ2.6c antigen [20]. Recently, it has been shown that GMZ2.6c is widely recognized by naturally acquired antibodies from individuals living in endemic areas of Brazil, and that levels of anti-GMZ2.6c antibodies increase with exposure to malaria and may contribute to immunity to the parasite. In addition, the higher prevalence of individuals with antibodies that recognize GMZ2.6c and the higher levels of anti-GMZ26c antibodies compared to its individual components suggest an additive effect of GLURP, MSP-3, and Pfs48/45 when inserted in the same construct [21]. Furthermore, the higher frequency and levels of antibodies against several linear B cell epitopes of GLURP, MSP-3, and Pfs48/45 indicate that the presence of these epitopes along the GMZ2.6c-chimeric protein may lead to a broad and robust immune response. These findings provide additional support for the relevance of GMZ2.6c as a multi-stage malaria vaccine candidate [22].

In addition to antibodies, cell-mediated responses are recognized as crucial for controlling parasites and providing protection against malaria. Malarial infections trigger antigen-specific CD8^+^ and CD4^+^ T cell responses upon contact with antigen-presenting cells (APCs), which present parasite antigens on Major Histocompatibility Complex (MHC) class I and II molecules, respectively, and provide costimulatory signals [23]. CD4^+^ T helper cells play a central role in regulating the immune response by producing pro- and anti-inflammatory cytokines, as well as activating macrophages and specific B cell clones [24]. Meanwhile, CD8^+^ T cells are the main effectors of the immune response against the intrahepatic stage, recognizing parasite-derived peptides presented by MHC class I on the surface of infected hepatocytes and Kupffer cells. Once activated, CD8^+^ T cells release effector mediators such as IFN-γ, which inhibits the development of malaria parasites, and granzymes and perforin, which induce host cell apoptosis through the caspase cascade and oxidative stress [25,26,27]. After the resolution of the infection, most CD4^+^ and CD8^+^ effector T cells undergo apoptosis, while induced memory T cells are maintained and capable of rapidly responding to secondary infection [23,28]. In this scenario, *P. falciparum* antigens that elicit antigen-specific T cell recall in naturally exposed populations could be considered for the development of a malaria vaccine that promotes long-lasting immunity.

T cell responses depend on the recognition of immunodominant peptides by MHC molecules. The main obstacle to identifying T cell epitopes is that the genes encoding Human Leukocyte Antigen (HLA) class I and II molecules are highly polymorphic and under selective pressure by malaria parasites in endemic areas [29]. The allelic forms of HLA present different binding affinities, which affect peptide-HLA complex formation and subsequent recognition by T cell receptors [30]. Therefore, it is crucial to identify immunogenic and promiscuous T cell epitopes that interact with multiple HLA molecules. The goal of this work was to evaluate the cellular immune response to the chimeric protein GMZ2.6c and its components (MSP-3, GLURP, and Pfs48/45) in individuals naturally exposed to malaria in the Brazilian Amazon and to identify and validate its promiscuous T cell epitopes.

## 2. Materials and Methods

### 2.1. Study Area and Volunteers

A cross-sectional study was carried out between July and August 2018 in three municipalities located in the Juruá Valley, a region of the Brazilian Amazon. The sites included Cruzeiro do Sul (07°37′50″ S/72°40′13″ W) and Mâncio Lima (07°36′49″ S/72°53′47″ W) in the state of Acre, and Guajará (07°54′85″ S/72°58′88″ W) in the state of Amazonas, all considered high-risk areas for *Plasmodium falciparum* infection. In Brazil, malaria transmission intensity is classified according to the Annual Parasitological Index (API), expressed as the number of autochthonous cases per 1000 inhabitants, and categorized as high (≥50), medium (≥10 and <50), low (>1 and <10), or very low (<1). In 2018, the API values were 147.4 for Cruzeiro do Sul, 422.8 for Mâncio Lima, and 124.5 for Guajará, with corresponding *P. falciparum* case reports of 2817, 1616, and 429, respectively [31].

Peripheral blood mononuclear cells (PBMCs) were obtained from individuals naturally exposed to malaria, either infected with *P. falciparum* (PFI group) or non-infected at the time of sampling (NI group). Additionally, PBMCs from 10 members of the laboratory staff (Rio de Janeiro, Brazil), with no history of malaria or contact with malaria-endemic areas, were included in our study as a non-endemic control group (Control).

### 2.2. Epidemiological Survey, Blood Sampling, Malaria Diagnosis, and PBMC Isolation

Participants who agreed to participate signed an informed consent form and completed an epidemiological survey. To assess the degree of malaria exposure, the survey collected information on age, time of residence in the endemic area, number of previous malaria episodes, time since the last infection, use of malaria prophylaxis, and presence of symptoms. Subsequently, 20 mL of venous peripheral blood was collected into heparin and EDTA tubes (Becton Dickinson, San Diego, CA, USA) for cellular immunity assays and molecular diagnosis, respectively.

Blood collected into EDTA tubes was mixed with an equal volume of a cryopreservation solution (0.9% NaCl/4.2% sorbitol/20% glycerol) and stored at −70 °C for subsequent DNA extraction. Thin and thick blood smears were prepared for parasitological diagnosis and examined by a technician experienced in malaria diagnosis at the Laboratório de Pesquisa em Malária (Fiocruz) headquarters of the CPD-Mal (Centro de Pesquisa, Diagnóstico e Treinamento em Malária), a reference center for malaria diagnosis for the Brazilian Ministry of Health. Malaria diagnosis was performed in Giemsa-stained thin and thick blood smears, with a parasitological evaluation involving the examination of 200 fields at 1000× magnification under oil immersion. Thin blood smears of positive samples were examined for species identification. To increase diagnostic sensitivity, molecular testing was performed on all samples. DNA was extracted using the QIAamp DNA Blood Mini Kit (Qiagen, Germantown, MD, USA), following the manufacturer’s protocol. PCR assays were carried out using genus-specific (*Plasmodium* sp.) and species-specific (*P. falciparum* and *P. vivax*) primers, as previously reported [32]. Donors diagnosed with *P. vivax* and/or *P. falciparum* infection at the time of sampling were treated according to the therapeutic guidelines established by the Brazilian Ministry of Health [33].

Heparinized blood samples were centrifuged for 10 min at 250× *g*. After removing the plasma, an equal volume of phosphate-buffered saline (PBS) was added. PBMCs were then isolated by density gradient centrifugation using Ficoll-Hypaque 1077 (Sigma, St. Louis, MO, USA) and washed twice with ice-cold PBS. The PBMCs were suspended in fetal bovine serum (FBS) (Gibco, Thermo Fisher Scientific Inc., Waltham, MA, USA) containing 10% dimethyl sulfoxide (Sigma, St. Louis, MO, USA) at 4 °C. The suspension was transferred into cryotubes, placed in a Nalgene “Mr. Frosty” freezing container, and stored overnight at −70 °C. The samples were subsequently stored in a liquid nitrogen tank until further use.

### 2.3. T Cell Epitope Prediction and Synthesis of Peptides

The prediction of potential T cell epitopes for GLURP_27–500_ (UniProt: Q8IJ56), MSP-3_155–249_ (UniProt: Q8IJ55), and Pfs48/45_291–428_ (UniProt: Q8I6T1) was conducted using the binding prediction tools provided by the Immune Epitope Database and Analysis Resource (IEDB) [34]. This resource utilizes various algorithms, including Artificial Neural Network (ANN) [35], Stabilized Matrix Method (SMM) [36], and Combinatorial Peptide Libraries (Comblib) [37]. The lengths of the predicted epitopes were 9 amino acids for MHC class I and 15 amino acids for MHC class II. The prediction scores were evaluated considering the most frequent HLA class I (HLA-A*01:01; HLA-A*02:01; HLA-A*02:03; HLA-A*02:06; HLA-A*03:01; HLA-A*11:01; HLA-A*23:01; HLA-A*24:02; HLA-A*26:01; HLA-A*30:01; HLA-A*30:02; HLA-A*31:01; HLA-A*32:01; HLA-A*33:01; HLA-A*68:01; HLA-A*68:02; HLA-B*07:02; HLA-B*08:01; HLA-B*15:01; HLA-B*35:01; HLA-B*40:01; HLA-B*44:02; HLA-B*44:03; HLA-B* 51:01; HLA-B*53:01; HLA-B*57:01; HLA-B*58:01) and class II (HLA-DRB1*01:01; HLA-DRB1*03:01; HLA-DRB1*04:01; HLA-DRB1*04:05; HLA-DRB1*07:01; HLA-DRB*08:02; HLA-DRB1*09:01; HLA-DRB1*11:01; HLA-DRB*12:01; HLA-DRB1*13:02; HLA-DRB1*15:01; HLA-DRB3*01:01; HLA-DRB3*02:02; HLA-DRB4*01:01; HLA-DRB5*01:01; HLA-DQA1*05:01/DQB1*02:01; HLA-DQA1*05:01/DQB1*03:01; HLA-DQA1*03:01/DQB1*03:02; HLA-DQA1*04:01/DQB1*04:02; HLA-DQA1*01:01/DQB1*05:01; HLA-DQA1*01:02/DQB1*06:02; HLA-DPA1*02:01/DPB1*01:01; HLA-DPA1*01:03/DPB1*02:01; HLA-DPA1*01:03/DPB1*04:01; HLA-DPA1*03:01/DPB*04:02; HLA-DPA1*02:01/DPB1*05:01; HLA-DPA1*02:01/DPB1*14:01) alleles worldwide. Lengths with a mean consensus score of less than 20 and at least 50% HLA binding frequency were considered potential T cell epitopes.

Peptide sequences corresponding to predicted T cell epitopes from the regions MSP-3_155–249_, GLURP_27–500_, and Pfs48/45_291–428_ were synthesized by GenOne Biotechnologies (Rio de Janeiro, RJ, Brazil) using fluorenylmethoxycarbonyl (Fmoc) solid-phase synthesis. Analytical chromatographic evaluation confirmed that all peptides exhibited purity levels exceeding 95%.

### 2.4. Recombinant Proteins

The multi-stage GMZ2.6c construct was generated by fusing the GLURP_79–1500_ and MSP-3_462–747_ to Pfs48/45_859–1284_ (region 6c). Expression of GMZ2.6c and its fragments was carried out in *Lactococcus lactis* MG1363, followed by purification as previously reported [20]. Briefly, *L. lactis* containing pSS4 was cultured in LAB medium supplemented with 5 mM cysteamine and 0.5 mM cystamine. The recombinant protein was then purified from the culture supernatant using affinity chromatography with a 5 mL HisTrap^TM^ HP column (GE Healthcare, Danderyd, Sweden), followed by a 5 mL HiTrap NHS-activated HP column containing monoclonal antibody mAb45.1 (epitope I), according to the manufacturer (GE Healthcare, Danderyd, Sweden). Protein purity was assessed by reversed-phase high-performance liquid chromatography (RP-HPLC), showing a relative purity > 95%. The production of GLURP_27–500_, MSP-3_183–354_, and Pfs48/45_291–428_ was performed according to established protocols [38,39].

### 2.5. PBMC Stimulation Assay

PBMCs were thawed in a 37 °C water bath and washed twice with RPMI-1640 medium (Sigma, St. Louis, MO, USA) containing 10% FBS (Thermo Fisher Scientific, Waltham, MA, USA) at 250× *g* for 10 min. Trypan blue viability of thawed PBMCs was >92% prior to culture. PBMCs were suspended in complete RPMI-1640 medium (10 mM Hepes, 1 mM sodium pyruvate, 200 U/mL penicillin, 200 µg/mL streptomycin, 55 µM 2-mercaptoethanol, and 2 g/L sodium bicarbonate), supplemented with 10% FBS. PBMCs were seeded in duplicate at 2.5 × 10^5^ cells per well in 96-well flat-bottom plates (Corning Inc., Corning, NY, USA) in a final volume of 200 µL of complete RPMI-1640 medium alone (unstimulated control), with PMA (50 ng/mL) and Ionomicin (250 ng/mL) as positive stimulation control, or with GMZ2.6c, GLURP, MSP-3, or Pfs48/45 (10 μg/mL). After incubation at 37 °C in 5% CO_2_ for 96 h, the culture supernatants were stored at −70 °C, and the cells were stained for flow cytometry analysis.

### 2.6. Immunophenotyping of Lymphocyte Subpopulations and Cellular Activation

PBMCs (both ex vivo and after stimulation) were initially stained with monoclonal antibodies targeting cell surface molecules for 40 min at 4 °C in the dark (Table S1). After washing, PBMCs were incubated with Annexin V buffer containing Annexin V and 7-Aminoactinomycin D (7-AAD) (BD Bioscience, San Diego, CA, USA) for 15 min at 4 °C in the dark to gate viable lymphocytes (AnnexinV^−^/7-AAD^−^). The cells were resuspended in Annexin V buffer and immediately acquired using the CytoFlex flow cytometer (Beckman Coulter, Indianapolis, IN, USA) at the Flow Cytometry Platform of the Instituto Oswaldo Cruz. The data were analyzed using FlowJo software v10 (Tree Star Inc., Ashland, OR, USA). A minimum of 50,000 lymphocyte-gated events were evaluated based on scatter parameters of size and granularity. The gating strategies for viability, T cell activation, and memory T cell panels are illustrated in Figure S1.

### 2.7. ELISpot Assay

ELISpot assays were carried out using the commercial kit ELISpot Plus: Human IFN-γ (ALP) (MabTech, Nacka Strand, Sweden) according to the manufacturer’s instructions. Briefly, cell cultures were performed in duplicate on nitrocellulose 96-well plates pre-coated with anti-IFN-γ monoclonal antibody (Clone D1K). The plates were blocked with RPMI-1640 medium (Sigma, St. Louis, MO, USA) supplemented with 10% FBS for 30 min and washed four times with PBS. PBMCs were added at a concentration of 2.5× 10^5^ cells per well along with RPMI-1640 medium alone, 10 μg/mL of each peptide pool, or CD3-2 monoclonal antibody as a positive control. The cells were stimulated for 24 h at 37 °C in an atmosphere of 5% CO_2_. After stimulation, the plates were washed four times with PBS and incubated with 1 μg/mL of anti-human IFN-γ-biotin (Clone 7-B6-1) diluted in PBS containing 0.5% FBS for 90 min at 37 °C. The plates were washed four times with PBS and incubated with streptavidin-alkaline phosphatase diluted 1:1000 in PBS containing 0.5% FBS for 1 h at room temperature. After another four washes with PBS, the plates were developed with 1-step NBT/BCIP. The development was stopped by washing the membrane with distilled water. Spots formed by IFN-γ-secreting cells were scanned and counted using the Immunospot S6UV Ultra Analyzer (Cellular Technology Ltd., Cleveland, OH, USA). The results were expressed as spot-forming units (SFU) per 2.5 × 10^5^ cells. Individuals were considered responders if the mean number of SFU in the peptide-stimulated wells, after subtracting the mean SFU in the wells with medium alone, was greater than 20 SFU/2.5 × 10^5^ cells.

### 2.8. Cytokine Detection

The concentrations of IL-2, IL-10, TNF, IL-4, IFN-γ, IL-6, and IL-17A cytokines in culture supernatants were measured using the BD Cytometric Bead Array (CBA) Human Th1/Th2/Th17 kit (BD Bioscience, San Diego, CA, USA), following the manufacturer’s protocol. Briefly, the captured beads coated with cytokine-specific antibodies were mixed with cytokine standards or supernatant samples and incubated with phycoerythrin-conjugated detection antibody for 3 h at room temperature in the dark. After incubation, samples were washed and resuspended in a wash buffer, and acquired using the CytoFlex flow cytometer (Beckman Coulter, Indianapolis, IN, USA) at the Flow Cytometry Platform of the Instituto Oswaldo Cruz. Cytokine concentrations were determined using FCAP Array Software (BD Biosciences, San Diego, CA, USA). 

### 2.9. Statistical Analysis

Data were recorded in the Epi-Info v6 database (Centers for Disease Control and Prevention, Atlanta, GA, USA) and analyzed using both Epi-Info v6 and GraphPad Prism v9 (GraphPad Software Inc., San Diego, CA, USA). The distribution of variables was assessed for normality using the one-sample Kolmogorov–Smirnov test. For multiple comparisons, one-way ANOVA followed by Tukey’s post hoc test was applied, while Student’s *t*-test was used to evaluate the differences in mean values between the two groups for parametric variables. For non-parametric variables, the Kruskal–Wallis test followed by Dunn’s test was employed for multiple comparisons, and the Mann–Whitney test was used to assess differences in distributions. The chi-square test was used to analyze differences between the proportions of responders. A two-sided *p*-value of ≤0.05 was considered statistically significant.

## 3. Results

### 3.1. Characteristics of the Studied Population

The studied population consisted of 20 *P. falciparum*-infected and 42 non-infected individuals living in three malaria-endemic areas of the Brazilian Amazon. Additionally, 10 members of the laboratory staff (Rio de Janeiro, Brazil), who had neither a history of malaria nor contact with malaria transmission areas, were included as a non-exposed control group. The main characteristics of the studied population are shown in Table 1. There were no significant differences between the *P. falciparum*-infected (PFI) and non-infected (NI) groups in terms of gender, age, number of past malaria episodes, time since the last malaria episode, or species involved in the last malaria episodes. However, the NI group had a higher time of residence in malaria-endemic areas than the PFI group (*p =* 0.03), representing a longer natural exposure to malaria infections. Additionally, the control group showed a different gender distribution than the PFI group, with a higher proportion of females (*p* = 0.02) and a lower average age than the NI group (*p* = 0.02).

### 3.2. Ex Vivo Lymphocyte Subsets and Cellular Activation

**Figure 1:**
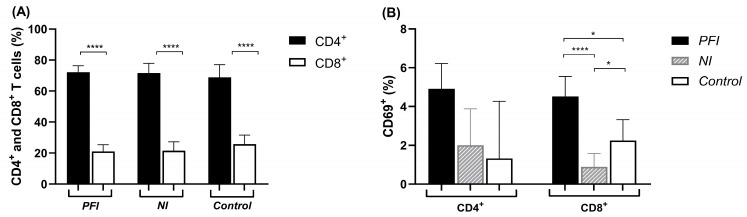
Ex vivo CD4^+^ and CD8^+^ T cell frequency and activation state. Analysis of CD4^+^ and CD8^+^ T cell frequency (**A**) and cellular activation based on CD69 expression (**B**) in *P. falciparum*-infected (PFI, n = 20), non-infected (NI, n = 20), and non-endemic control (Control, n = 10) groups ex vivo. Bars represent means, and lines represent standard deviations. Statistical significance was calculated using Student’s *t*-test and one-way ANOVA with Tukey’s post hoc test. Significant differences are indicated by * (* *p* < 0.05; **** *p* < 0.0001).

The phenotypic analysis of ex vivo PBMCs revealed that CD4^+^ T cells were more prevalent than CD8^+^ T cells across all groups (*p* < 0.0001) (Figure 1A). No significant differences were found when comparing CD4^+^ and CD8^+^ T cells among the three studied groups. Lymphocyte activation was determined by measuring the expression of the CD69 marker. The PFI group presented a higher percentage of CD8^+^CD69^+^ T cells compared to the NI and control groups (NI: *p* < 0.0001; Control: *p* = 0.04). Although no significant difference was observed in the percentage of CD4^+^CD69^+^ T cells, these cells also tended to be more frequent in the PFI group (Figure 1B).

**Figure 2:**
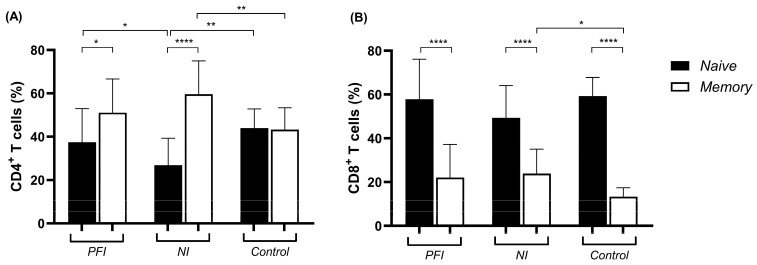
Frequency of naive and memory CD4^+^ and CD8^+^ T cells ex vivo. Flow cytometry analysis of CD4^+^ (**A**) and CD8^+^ (**B**) naive and memory T cells from exposed *P. falciparum*-infected (PFI, n *=* 20), non-infected (NI, n *=* 20), and non-endemic control (Control, n = 10) groups ex vivo. Bars represent means, and lines represent standard deviations. Statistical significance was calculated using Student’s *t*-test and one-way ANOVA with Tukey’s post hoc test. Significant differences are indicated by * (* *p* < 0.05; ** *p* < 0.005; **** *p* < 0.0001).

To determine the phenotype of CD4^+^ and CD8^+^ T cell subsets, we first characterized the naive (CD45RA^+^CD45RO^−^) and memory (CD45RA^−^CD45RO^+^) cells based on the expression of the different isoforms of the common leukocyte antigen CD45. Analysis of CD4^+^ T cells showed that PFI (*p* = 0.009) and NI (*p* < 0.0001) groups presented a higher percentage of memory than naive cells, while no difference was observed in the control group. The NI group showed a lower frequency of naive CD4^+^ T cells compared to both the PFI (*p* = 0.02) and control (*p* = 0.0006) groups (Figure 2A). For CD8^+^ T cells, all three groups studied exhibited higher frequencies of naive compared to memory cells (*p* < 0.0001) (Figure 2B). Additionally, lower percentages of memory CD4^+^ (*p* = 0.005) and CD8^+^ (*p* = 0.007) T cells were observed in the control group when compared to the NI group (Figure 2).

**Figure 3:**
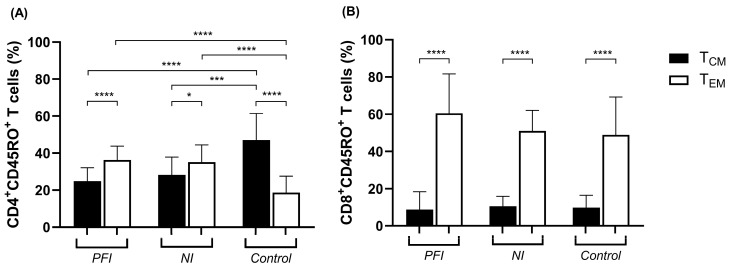
Subsets of memory CD4^+^ and CD8^+^ T cells ex vivo. Flow cytometry analysis of CD4^+^ (**A**) and CD8^+^ (**B**) central (T_CM_) and effector (T_EM_) memory T cells from exposed *P. falciparum*-infected (PFI, n = 20), non-infected (NI, n = 20), and non-endemic control (Control, n = 10) groups ex vivo. Bars represent means, and lines represent standard deviations. Statistical significance was calculated using Student’s *t*-test and one-way ANOVA with Tukey’s post hoc test. Significant differences are indicated by * (* *p* < 0.05; *** *p* < 0.0005; **** *p* < 0.0001).

Based on the expression patterns of CC-chemokine receptor 7 (CCR7) and L-selectin (CD62L) surface markers, memory T cells can be categorized into central memory (T_CM_) (CCR7^+^CD62L^+^) and effector memory (T_EM_) (CCR7^−^CD62L^−^) cells. Our findings indicated that the PFI (*p* < 0.0001) and NI (*p* = 0.02) groups presented higher percentages of CD4^+^ T_EM_ than T_CM_ cells, whereas the control group showed the opposite pattern, in which CD4^+^ T_CM_ cells were more frequent than CD4^+^ T_EM_ cells (*p* < 0.0001). Consequently, the control group showed higher percentages of T_CM_ and lower percentages of T_EM_ cells compared to both the PFI (*p* < 0.0001, for both) and NI (*p* = 0.0002 and *p* < 0.0001, for T_CM_ and T_EM_, respectively) groups (Figure 3A). All groups presented higher percentages of CD8^+^ T_EM_ cells than T_CM_ cells (*p* < 0.0001), with no significant differences observed between groups (Figure 3B).

### 3.3. T Cell Immune Response to GMZ2.6c and Its Components

PBMCs from PFI, NI, and control groups were incubated with GMZ2.6c, GLURP, MSP-3, and Pfs48/45, and the specific response was assessed. In all analyses, data were normalized using unstimulated samples as a baseline and expressed as percentages relative to unstimulated samples (Unstimulated: 100%). The control group showed no significant differences between unstimulated PBMCs and those stimulated with *P. falciparum* antigens.

**Figure 4:**
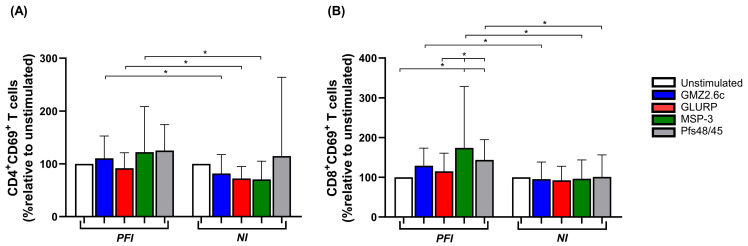
T cell activation after GMZ2.6c, GLURP, MSP-3, and Pfs48/45 antigen stimulation. Analysis of CD4^+^ (**A**) and CD8^+^ (**B**) T cell activation from *P. falciparum*-infected (PFI, n = 20) and non-infected (NI, n = 20) exposed groups following stimulation with GMZ2.6c, GLURP, MSP-3, and Pfs48/45 antigens. Unstimulated PBMCs were used as a baseline to normalize the percentages of activated lymphocytes (Unstimulated: 100%). Bars represent medians, and lines represent interquartile ranges. Statistical significance was calculated using Mann–Whitney test and Kruskal–Wallis test, followed by Dunn’s post hoc test. Significant differences are indicated by * (* *p* < 0.05).

No significant differences were observed in the frequencies of CD4^+^ and CD8^+^ T cells (Figure S2) or CD4^+^ T cell activation between the stimuli in the studied groups (Figure 4A). However, in the PFI group, a higher percentage of activated CD8^+^ T cells was noted following stimulation with MSP-3 and Pfs48/45 compared to unstimulated PBMCs (MSP-3: *p* = 0.01; Pfs48/45: *p* = 0.005), as well as compared to GLURP stimulation (MSP-3: *p* = 0.04; Pfs48/45: *p* = 0.02), whereas no differences in CD8^+^ T cell activation were observed in the NI group (Figure 4B). Moreover, when comparing PFI and NI groups, higher percentages of activated CD4^+^ T cells in response to GMZ2.6c (*p* = 0.02), GLURP (*p* = 0.02), and MSP-3 (*p* = 0.01), as well as activated CD8^+^ T cells in response to GMZ2.6c (*p* = 0.01), MSP-3 (*p* = 0.03), and Pfs48/45 (*p* = 0.01), were observed in the PFI group (Figure 4A,B).

**Figure 5:**
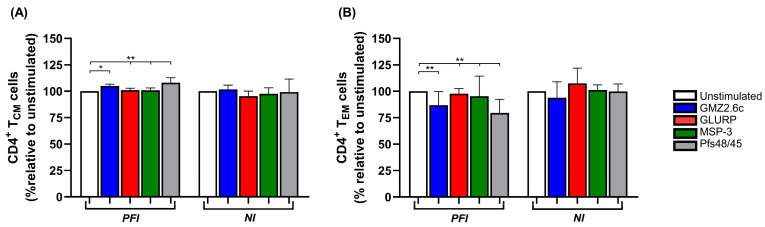
Subsets of memory CD4^+^ T cells after GMZ2.6c, GLURP, MSP-3, and Pfs48/45 antigen stimulation. Analysis of central (T_CM_) (**A**) and effector (T_EM_) (**B**) memory CD4^+^ T cells from exposed *P. falciparum*-infected (PFI, n = 20) and non-infected (NI, n = 20) groups after stimulation with GMZ2.6c, GLURP, MSP-3, and Pfs48/45 antigens. Unstimulated PBMCs were used as a baseline to normalize the percentages of stimulated PBMCs (Unstimulated: 100%). Bars represent medians, and lines represent interquartile ranges. Statistical significance was calculated using Mann–Whitney test and Kruskal–Wallis test, followed by Dunn’s post hoc test. Significant differences are indicated by * (* *p* < 0.05; ** *p* < 0.005).

Regarding the functional phenotype of CD4^+^ and CD8^+^ T cells, no significant differences were observed in the percentages of naive and memory T cells among the different stimuli (Figure S2), as they showed similar proportions to those found ex vivo. However, when the frequency of memory T cells was analyzed in the PFI group, an increase in CD4^+^ T_CM_ cells and a reduction in CD4^+^ T_EM_ cells were observed after stimulation with Pfs48/45 (Unstimulated: *p* < 0.0001 for both; GLURP: T_CM_: *p* = 0.002, T_EM_: *p* = 0.004; MSP-3: T_CM_: *p* = 0.003, T_EM_: *p* = 0.008) and GMZ2.6c (Unstimulated: T_CM_: *p* = 0.01, T_EM_: *p* = 0.002) compared to unstimulated or GLURP- and MSP-3-stimulated PBMCs. No significant differences were observed in CD4^+^ T_CM_ and T_EM_ in the NI group, or in CD8^+^ T_CM_ and T_EM_ in either group (Figure 5A,B and Figure S2).

### 3.4. Cytokine Levels from Culture Supernatants

The concentrations of cytokines IL-2, IL-10, TNF, IL-4, IFN-γ, IL-6, and IL-17A secreted by PBMCs from the study participants were measured following stimulation with GMZ2.6c, GLURP, MSP-3, and Pfs48/45 antigens. Changes in the magnitude of cytokine production were calculated relative to unstimulated samples (Unstimulated: 100%).

**Figure 6:**
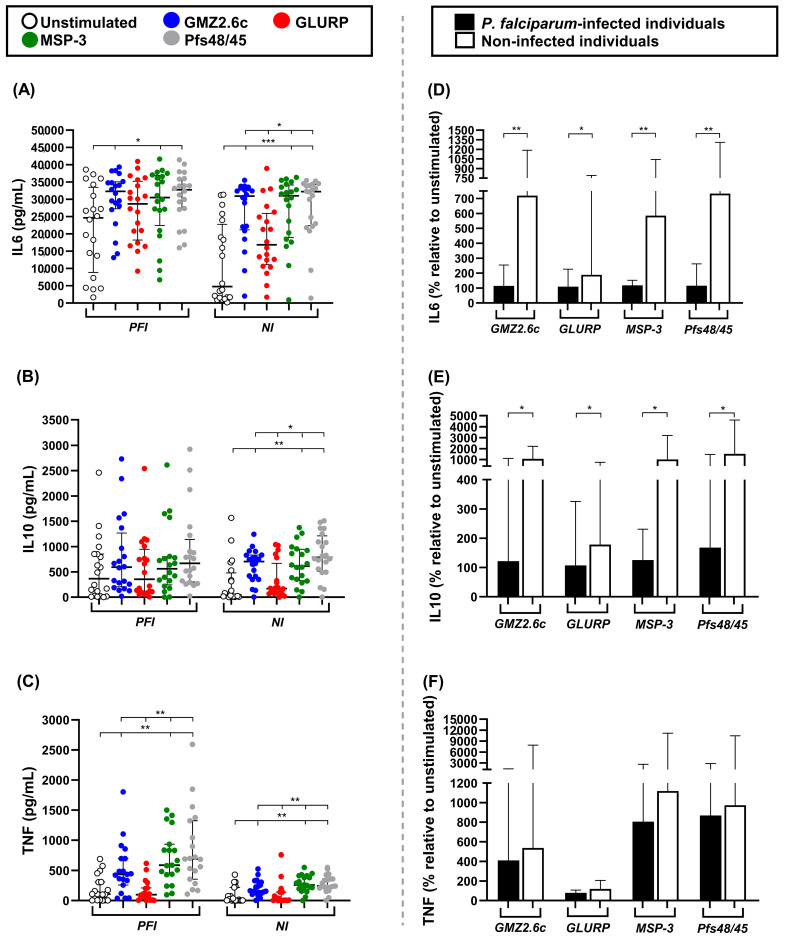
PBMC cytokine responses upon stimulation with GMZ2.6c, GLURP, MSP-3, and Pfs48/45. Concentrations (**A**–**C**) and changes in the magnitude (**D**–**F**) of IL-6, IL-10, and TNF in the supernatants of PBMC cultures from exposed *P. falciparum*-infected (PFI, n = 20) and non-infected (NI, n = 20) groups. Unstimulated PBMCs were used as a baseline to normalize the magnitude of cytokine responses after antigen stimulation (Unstimulated: 100%). Dots represent individual values, bars represent medians, and lines represent interquartile ranges. Statistical significance was calculated using Mann–Whitney test and Kruskal–Wallis test, followed by Dunn’s post hoc test. Significant differences are indicated by * (* *p* < 0.05; ** *p* < 0.005; *** *p* < 0.0005).

The concentrations of IL-4 were below the detection limit (4.9 pg/mL) and were not included in the statistical analysis. The concentrations of IL-2, IFN-γ, and IL-17A did not differ significantly between unstimulated PBMCs and those stimulated with *P. falciparum* antigens (Figure S3). The PFI and NI groups showed higher concentrations of IL-6 after stimulation with GMZ2.6c (PFI: *p* = 0.02; NI: *p* = 0.0001), MSP-3 (PFI: *p* = 0.03; NI: *p* < 0.0001), and Pfs48/45 (PFI: *p* = 0.01; NI: *p* < 0.0001) compared to unstimulated PBMCs. In the NI group, PBMCs stimulated with GMZ2.6c (*p* = 0.02), MSP-3 (*p* = 0.01), and Pfs48/45 (*p* = 0.004) also produced higher concentrations of IL-6 than those stimulated with GLURP (Figure 6A). No significant differences in IL-10 concentrations were observed in the PFI group. However, the NI group exhibited higher concentrations of IL-10 after stimulation with GMZ2.6c, MSP-3, and Pfs48/45 compared to unstimulated PBMCs (GMZ2.6c: *p* = 0.002; MSP-3: *p* = 0.001; Pfs48/45: *p* < 0.0001) and to PBMCs stimulated with GLURP (GMZ2.6c: *p* = 0.03; MSP-3: *p* = 0.02; Pfs48/45: *p* = 0.001) (Figure 6B). The PFI and NI groups showed higher TNF concentrations after stimulation with GMZ2.6c, MSP-3, and Pfs48/45 compared to unstimulated PBMCs (PFI: *p* = 0.006, *p* < 0.0001, and *p* < 0.0001 for GMZ2.6c, MSP-3, and Pfs48/45, respectively; NI: *p* = 0.007, *p* < 0.0001, and *p* < 0.0001 for GMZ2.6c, GLURP, and Pfs48/45, respectively) or with GLURP-stimulated PBMCs (PFI: *p* = 0.002, *p* < 0.0001, and *p* < 0.0001 for GMZ2.6c, MSP-3, and Pfs48/45, respectively; NI: *p* = 0.007, *p* < 0.0001, *p* < 0.0001 for GMZ2.6c, MSP-3, and Pfs48/45, respectively) (Figure 6C). The magnitude of cytokine responses was compared between NI and PFI groups, revealing increased production of both IL-6 (GMZ2.6c: *p* = 0.009; GLURP: *p* = 0.01; MSP-3: *p* = 0.008; Pfs48/45: *p* = 0.009) and IL-10 (GMZ2.6c: *p* = 0.01; GLURP: *p* = 0.04; MSP-3: *p* = 0.02; Pfs48/45: *p* = 0.01) in the NI group (Figure 6D–F).

### 3.5. Prediction and IFN-γ Responses to MSP-3, GLURP, and Pfs48/45 Peptides

Using the IEDB binding prediction tools, sequences within MSP-3_155–249_, GLURP_27–500_, and Pfs48/45_291–428_ were identified as potential CD4 or CD8 T cell epitopes and organized into four different pools for use in ELISpot assays (Table 2).

**Figure 7:**
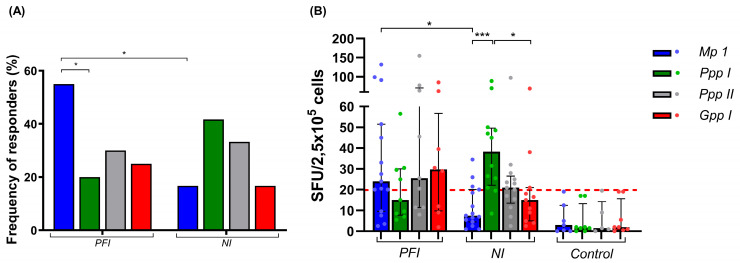
IFN-γ responses to MSP-3, GLURP, and Pfs48/45 synthetic peptides. Frequency of positive responders (**A**) and numbers of spots-forming units (SFU) (**B**) of IFN-γ responses to Mp 1 (blue), Ppp I (green), Ppp II (gray), and Gpp I (red) in exposed *P. falciparum*-infected (PFI, n = 20), non-infected (NI, n = 25), and non-endemic control (Control, n = 10) individuals. Bars represent the frequency of responders (**A**) and medians of adjusted SFU of IFN-γ responses to each peptide pool (**B**). Lines represent the interquartile ranges. Dashed red lines represent the positivity limit. Statistical significance was calculated using chi-square, Mann–Whitney test, and Kruskal–Wallis test, followed by Dunn’s post hoc test. Significant differences are indicated by * (* *p* < 0.05; *** *p* < 0.0005).

To validate the predictions and evaluate the antigenicity of potential T cell epitopes, PBMCs from 20 *P. falciparum*-infected (PFI), 25 non-infected (NI), and 10 non-endemic control (Control) individuals were used. Our data showed that all peptide pools induced an IFN-γ response in both PFI and NI groups, with the frequency of responders to Mp1, Ppp I, Ppp II, and Gpp I of 55% (11/20), 20% (4/20), 30% (6/20), and 25% (5/20), in the PFI group, and 16.7% (4/24), 41.7% (10/24), 33.3% (8/24), and 16.7% (4/24) in the NI group, respectively. In the PFI group, the Mp1 peptide was recognized more frequently than Ppp I (*p* = 0.02), whereas no significant differences were observed in the NI group (Figure 7A). In the NI group, the median of adjusted IFN-γ spots-forming units (SFU) per 2.5 × 10^5^ cells elicited by Ppp I was higher than that for Mp 1 (*p =* 0.0003) and Gpp I (*p =* 0.009), while no statistical differences were observed in the PFI group (Figure 7B). When comparing both groups, a higher frequency of responders (*p =* 0.007) and SFU (*p =* 0.006) against Mp1 was observed in the PFI group. None of the 10 non-endemic control individuals demonstrated significant IFN-γ responses to any of the four peptide pools tested (Figure 7).

## 4. Discussion

The GMZ2.6c chimeric protein is a *P. falciparum* malaria vaccine candidate developed based on studies suggesting that GMZ2 and Pfs48/45 are promising antigens for blood stage and transmission-blocking vaccines, respectively [40,41,42,43,44,45,46,47,48,49,50]. Previously, we characterized the naturally acquired humoral immune response to GMZ2.6c, showing that GMZ2.6c is widely recognized by antibodies from individuals living in endemic areas of the Brazilian Amazon and that the levels of these antibodies appear to increase with exposure to malaria infection and may contribute to immunity against the parasite [21]. In the present study, we focus on characterizing T cell immune response profiles to the GMZ2.6c, its individual components (MSP-3, GLURP, and Pfs48/45), and synthetic peptides in individuals from malaria-endemic areas of Brazil.

Studies using experimental murine models and human subjects have highlighted the critical role of antigen-specific CD4^+^ and CD8^+^ T cells in protective responses to blood stage malaria [51,52,53,54]. Thus, we evaluated CD4^+^ and CD8^+^ T cell responses ex vivo and after in vitro stimulation with GMZ2.6c, GLURP, MSP-3, and Pfs48/45, considering the activation and functional profiles of these cell subsets. Individuals living in Brazilian malaria-endemic areas, whether non-infected or infected with *P. falciparum*, showed no significant differences in the percentages of CD4^+^ and CD8^+^ T cells, both ex vivo and after culture in the absence or presence of *P. falciparum* antigens. This finding contrasts with several studies reporting a reduction in the percentage or absolute number of T cells in peripheral blood during acute *P. falciparum* or *P. vivax* infection, attributed to apoptosis or reallocation of these cells to sites of inflammation [55,56,57,58]. Moreover, no differences were observed in CD4^+^ T cell activation after culture in the absence or presence of *P. falciparum* antigens. These results are consistent with previous studies demonstrating that exposed individuals immunized with GMZ2 showed no significant changes in the proportion of total CD4^+^ T cells or in the frequency of CD4^+^ T cells secreting pro- and anti-inflammatory cytokines before and after immunization [50]. Additionally, the PfSPZ vaccine was found to be less immunogenic in malaria-exposed adults than in children [59], suggesting that continuous natural exposure to the parasite may impair sustained T cell activation and/or affect dendritic cell priming [60]. Curiously, the exposed *P. falciparum*-infected group, but not the non-infected group, showed increased CD8^+^ T cell activation upon stimulation with MSP-3 and Pfs48/45.

CD8^+^ T lymphocytes are known to eliminate infected hepatocytes through the release of granzyme B and perforins [23,25], as well as to inhibit intrahepatic parasite development by producing IFN-γ and TNF [27]. Specific CD8^+^ T lymphocytes against pre-erythrocytic antigens have also been described in the blood of individuals living in malaria-endemic areas [61,62] and after vaccination [63,64]. Although CD8^+^ T cells play a protective role against the sporozoite and liver stages, studies have reported conflicting evidence regarding their function during the blood stage of the parasite’s lifecycle. In experimental rodent models, acute blood stage infection triggers a robust and functional CD8^+^ T cell response that has been associated with either high parasite burdens and cerebral malaria immunopathogenesis [65,66,67,68,69] or, conversely, with parasite control and long-term immunity against blood stage disease [70,71]. However, in humans, naive donors submitted to experimental *P. falciparum* infection showed low IFN-γ-producing CD8^+^ T cell recall responses to asexual blood stage parasites [72], and in individuals with lifelong exposure to malaria, decreased responses to *P. falciparum* schizont extract have been associated with the suppressive effect of CD8^+^ T cells [73]. Our findings indicate that during *P. falciparum* infection, specific CD8^+^ T cells targeting MSP-3 and Pfs48/45 are induced and, although they do not expand, they remain capable of recalling responses against these antigens upon in vitro restimulation. Nevertheless, the contribution of these cells to protective immunity remains unclear. Furthermore, the similar pattern of CD8^+^ T cell activation observed in the absence of antigen or following GMZ2.6c stimulation in the *P. falciparum*-exposed group suggests that the conformation of GMZ2.6c may influence epitope processing and subsequent presentation through MHC class I to CD8^+^ T lymphocytes. Indeed, the three-dimensional structure of *P. falciparum* MSP-1 has been shown to determine epitope immunodominance [74], and this modulation may result from proteolytic processing [75,76].

During malaria infection, antigen-specific CD4^+^ and CD8^+^ T cell responses are induced. Once the infection resolves, most effector cells undergo apoptosis, while memory T cells are retained, allowing a rapid response to subsequent infections [23,77]. A key objective of vaccine development is to identify antigens capable of inducing long-lasting immunological memory. Our results demonstrated similar profiles of naive and memory CD4^+^ and CD8^+^ T cells both ex vivo and after in vitro culture, regardless of the stimulus. Interestingly, both exposed *P. falciparum*-infected and non-infected groups showed no differences in memory CD4^+^ and CD8^+^ T cell subsets ex vivo, indicating the predominance of effector memory T cells even after acute *P. falciparum* infection, consistent with previous studies in rodent models [78,79]. However, exposed *P. falciparum*-infected individuals showed an increase in central memory and a reduction in effector memory CD4^+^ T cells after stimulation with GMZ2.6c and Pfs48/45. This result may be attributed to chronic stimulation, resulting in CD4^+^ T cell exhaustion, which is characterized by diminished proliferative capacity and reduced effector functions in response to parasite antigens [23,70,80].

Central memory T cells are known to provide protection against *P. falciparum* infection [81]. Notably, no significant differences were observed in the percentages of central and effector CD4^+^ and CD8^+^ T cell subsets within the exposed non-infected group, regardless of *P. falciparum* antigen stimulation. Similarly, in the *P. falciparum*-infected group, no differences were observed after stimulation with GLURP and MSP-3. Studies in experimental murine models and in individuals exposed to *P. falciparum* have shown comparable profiles of memory CD4^+^ and CD8^+^ T cell subsets in response to reinfection or chronic parasite exposure, although cytokine production by these cells may reflect functional differences [80,82,83]. In fact, specific effector memory CD4^+^ and CD8^+^ T cells producing IFN-γ against *P. falciparum* MSP-1 are associated with parasite exposure [84], whereas central memory T cells producing TNF-α and IL-2 are associated with T cell proliferation and reduced parasitemia [81].

Protective immune responses against malaria rely on a delicate balance between pro-inflammatory and anti-inflammatory cytokines [85,86,87]. Thus, we evaluated the cytokine profiles in the supernatant of cell culture stimulated with GMZ2.6c, GLURP, MSP-3, and Pfs48/45. Our results showed that cells stimulated with GMZ2.6c, MSP-3, and Pfs48/45 produced higher levels of IL-6 and TNF in the *P. falciparum*-infected group, and IL-6, TNF, and IL-10 in the exposed non-infected group. These findings suggest that the strong pro-inflammatory environment during acute *P. falciparum* infection may modulate subsequent responses to parasite antigens, promoting a Th1 profile characterized by IL-6 and TNF secretion.

Several studies have shown that pro-inflammatory cytokines play a crucial role in controlling parasite growth and in the protective immune response against malaria [77,88]. However, exacerbated levels of these cytokines have been associated with the pathogenesis and severity of *P. falciparum* malaria [89,90]. IL-6 is particularly important during the early stages of malaria infection, as it promotes the upregulation of Inducible T cell Costimulator (ICOS) on T follicular helper (Tfh) cells and supports B cell differentiation during blood stage infection, leading to the production of parasite-specific antibodies [88,91], and may also contribute to the naturally acquired antibody response against GMZ2.6c, MSP-3, and Pfs48/45, as observed in our previous study [21]. The simultaneous increase of IL-6, TNF, and IL-10 in the exposed non-infected group suggests a balance between pro- and anti-inflammatory cytokines. IL-10 is a key regulatory cytokine that maintains this balance by inhibiting the secretion of IFN-γ and TNF, preventing pathological effects associated with their continuous production, and is linked to protection from severe malaria in both humans and experimental models [92]. These data corroborate previous studies showing that stimulation with *P. vivax* MSP-1_19_ and *P. falciparum* Liver-Stage Antigen 1 (LSA-1) induced high IL-10 levels in individuals exposed to malaria in southern Thailand and Kenya, respectively [93,94]. Additionally, reexposure of African children to *P. falciparum* has been associated with the acquisition of parasite-specific IL-10 responses [95,96], suggesting regulation of the inflammatory response while enhancing anti-parasite effector mechanisms. Notably, the exposed non-infected group showed higher IL-6 and IL-10 levels after PBMC stimulation with GMZ2.6c, GLURP, MSP-3, and Pfs48/45 compared to the exposed *P. falciparum*-infected group, indicating that chronic exposure to *P. falciparum* can recall antigen-specific pro- and anti-inflammatory cytokine responses, whereas these responses may be diminished in infected individuals. Several studies have reported suppressed immune responses during *Plasmodium* infection in humans and murine models, including reduced lymphocyte numbers, upregulation of inhibitory markers, and increased circulation of functional polymorphonuclear myeloid-derived suppressor cells [70,83,97,98,99,100,101,102].

An additional aspect that may contribute to the modulation of cellular immune responses observed in our study is the role of regulatory T cells (Tregs). These cells are known to expand during malaria infection and can suppress effector T cell proliferation and cytokine production, thereby limiting parasite clearance but also preventing excessive immunopathology. Previous studies have shown that Tregs can modulate both CD4^+^ and CD8^+^ T cell responses after *Plasmodium* infection, potentially leading to reduced responsiveness upon antigen restimulation [103]. Although we did not specifically assess Tregs in our cohort, it is plausible that their activity contributed to the dampened cellular immunity observed. Future studies directly evaluating Treg frequency and function in response to GMZ2.6c and its components will be important to clarify their role in shaping naturally acquired immunity to malaria.

Together, these findings suggest that after natural *P. falciparum* infection, specific memory T cells targeting MSP-3 and Pfs48/45 are induced and may trigger cytokine production in response to GMZ2.6c stimulation. However, among the components of GMZ2.6c, GLURP did not recall T cell responses in individuals from Brazilian malaria-endemic areas. Considering that the induction of antibody-producing cells against protein antigens typically requires direct interaction between activated B cells and CD4^+^ T cells, these findings contrast with our previous study, which reported both a high frequency of responders and naturally acquired antibody levels against GLURP in the same population [21].

Identifying T cell epitopes capable of eliciting immune responses in individuals of diverse genetic backgrounds remains a major challenge in developing malaria subunit vaccines. Herein, twelve sequences were predicted as promiscuous HLA class I or II T cell epitopes and grouped into four pools for antigenic evaluation. Although we did not observe significant differences in IFN-γ concentrations in the supernatants of PBMC cultures with the recombinant proteins, all four pools induced an IFN-γ response in individuals previously exposed to *P. falciparum*, as detected by the sensitive ELISpot assay, regardless of parasite presence. These findings support previous studies indicating that prior malaria infections can lead to the development of antigen-specific T cells capable of eliciting recall responses in the absence of the parasite [62,104].

Interferon-gamma plays a crucial role in immunity against *Plasmodium* infection. During the early stages of the disease, IFN-γ contributes to the recruitment and activation of macrophages and natural killer cells, promoting initial control of parasite growth [105,106,107]. As infection progresses, IFN-γ produced by CD4^+^ Th1 cells is important for activating CD8^+^ T and B cells, as well as recruiting macrophages to enhance parasite clearance [27,108,109]. High concentrations of IFN-γ induced by Circumsporozoite protein (CSP), Thrombospondin-related adhesive protein (TRAP), LSA-1, Apical Membrane Antigen 1 (AMA-1), MSP-1, and MSP-3 have been associated with protection after vaccination or natural exposure to *Plasmodium* [94,110,111,112,113,114,115]. However, we were unable to demonstrate a clear association between specific IFN-γ-producing T cells in response to these peptide pools and protection or exposure markers. Further studies are needed to elucidate their contribution to the immune response against *P. falciparum*. In both exposed groups, similar frequencies of responders to Ppp I, Ppp II, and Gpp I were observed. However, the higher frequency of responders and numbers of SFU to Mp1 in the *P. falciparum*-infected group may reflect a booster effect in specific T cell clones in response to acute infection.

The Pfs48/45 protein is one of the best-characterized surface antigens found on the gametocyte and gamete of *P. falciparum* and has been extensively studied as a target for transmission-blocking antibodies [116,117,118]. Although several studies have shown that antibodies against Pfs48/45 are naturally acquired through exposure to the parasite and can mediate transmission-reducing activity, the cellular immune responses to this antigen remain poorly understood. Earlier studies suggested that lymphoproliferative and IFN-γ responses to purified Pfs48/45 were limited, short-lived, and possibly the result of cross-reactivity with unrelated pathogens rather than specific memory against Pfs48/45. Additionally, Pfs48/45 was found to mainly induce T-independent and transient IgG responses [119,120,121]. In contrast, our findings provide the first direct evidence that natural infection with *P. falciparum* induces antigen-specific T cells targeting Pfs48/45. These T cells demonstrated recall responses upon in vitro stimulation in both *P. falciparum*-infected individuals (evidenced by CD8^+^ T cell activation, modulation of CD4^+^ T cell memory subsets, and production of IL-6 and TNF in response to recombinant protein, as well as IFN-γ responses to peptide pools) and non-infected individuals (demonstrated by the production of IL-6, TNF, and IL-10 in response to recombinant protein, along with IFN-γ responses to peptide pools), suggesting that specific memory T cells are induced during natural infection. These findings are particularly important because antibody levels against Pfs48/45 can be influenced by γ-interferon-inducible lysosomal thiol reductase (GILT), an enzyme that reduces disulfide bonds during antigen processing. This process affects the generation of T cell epitopes and, consequently, modulates T-helper cell responses necessary for the development of specific B cell responses [122]. Collectively, these results suggest that effective and lasting transmission-blocking immunity may rely on a coordinated interplay between cellular and humoral immune responses.

## 5. Conclusions

In conclusion, our findings demonstrate that individuals living in malaria-endemic areas of Brazil develop naturally acquired cellular immune responses against MSP-3 and Pfs48/45, which can be recalled after GMZ2.6c stimulation. The differences observed between exposed groups suggest that *P. falciparum* infection may modulate these responses toward a pro-inflammatory Th1 profile, while non-infected individuals showed a more balanced Th1/Th2 cytokine production. Additionally, multiple promiscuous HLA class I and II epitopes within GLURP, MSP-3, and Pfs48/45 were identified as capable of recalling IFN-γ responses. Further studies are needed to fully ascertain the potential of GMZ2.6c in inducing cellular immune responses and its viability as a protective candidate vaccine against malaria.

## Figures and Tables

**Table 1:** The studied population’s personal, clinical, and epidemiological characteristics.

		NI (n = 42)	PFI (n = 20)	Control (n = 10)
Personal Data				
Gender	Male	21 (50%)	13 (65%)	2 (20%) ^a^
	Female	21 (50%)	7 (35%)	8 (80%) ^a^
Age (years)—Mean ± SD		38.4 ± 13.4	31.4 ± 14.3	27.8 ± 6.1 ^b^
Time of residence in malaria-endemic areas (years)—Mean ± SD		37.2 ± 14 ^c^	28.5 ± 15.5	NA
Clinical and Epidemiological Data				
Number of past malaria episodes—Mean ± SD		9.9 ± 7.4	13.8 ± 9.6	NA
Time elapsed since the last malaria episode (months)—Median (IQR)		24 (7–47.5)	12 (1–24)	NA
Species of the last malaria episode	*P. vivax*	22 (52.4%)	8 (40%)	NA
	*P. falciparum*	16 (38.1%)	8 (40%)	NA
	*P. vivax* and *P. falciparum*	1 (2.4%)	0 (0%)	NA
	Not reported	3 (7.1%)	4 (20%)	NA

Age, time of residence in malaria-endemic areas (years), number of past malaria episodes, and time of symptoms (days) before diagnosis are represented as mean ± standard deviation. Time elapsed since the last malaria episode (months) is represented as median (interquartile range). n: number; %: percentage; SD: Standard Deviation; IQR: Interquartile Range; NA: Not Applicable. Statistical significance was assessed using chi-square test, Student’s *t*-test, and Mann–Whitney test. ^a^ *p =* 0.02 Control versus PFI; ^b^ *p =* 0.02 Control versus NI; ^c^ *p =* 0.03 NI versus PFI.

**Table 2:** Amino acid sequence and relative position of predicted T cell epitopes.

Pool	Peptide	Sequence	Amino Acid Position
MSP-3 MHC-I	Mp 1	SSYDYILGWEF	188–198
Pfs48/45 MHC-I (Ppp I)	Pp 1	KSAYMTVTI	417–425
	Pp 2	HTFTDSLDISL	308–318
	Pp3	KLFGIVGSI	394–402
	Pp4	GSIPKTTSF	400–408
Pfs48/45 MHC-II (Ppp II)	Pp5	LEPSNIVYLDSQINIGDI	364–381
	Pp6	IIPDCFFQVYQPESEELE	348–365
GLURP MHC-I (Gpp I)	Gp 1	KVQNHFESL	146–154
	Gp 2	KSNKVQNHF	143–151
	Gp 3	LVSENVPSGL	81–90
	Gp 4	KQNSQIPSL	309–317
	Gp 5	ETNIQEQLY	292–300

Mp: MSP-3 peptide; Ppp I: Pfs48/45 MHC-I peptide pool; Ppp II: Pfs48/45 MHC-II peptide pool; Pp: Pfs48/45 peptide; Gpp I: GLURP MHC-I peptide pool; Gp: Glurp peptide.

## Data Availability

The datasets supporting the conclusions of this article are included within the article and its Supplementary Materials.

## Supplementary Materials

The following supporting information can be downloaded at: https://www.mdpi.com/article/10.3390/vaccines14050423/s1, Table S1: Flow cytometry staining panels; Figure S1: Flow cytometry gating strategy for viability, T cell activation, and memory T cell panels; Figure S2: Lymphocyte subpopulations after GMZ2.6c, GLURP, MSP-3, and Pfs48/45 antigen stimulation; Figure S3: PBMC cytokine response under stimulation with GMZ2.6c, GLURP, MSP-3, and Pfs48/45.
