# Supplementary material for: Cellular Immune Response and T Cell Epitope Mapping of Plasmodium falciparum Chimeric Vaccine Candidate GMZ2.6c and Its Components (MSP-3, GLURP and Pfs48/45) in Individuals Naturally Exposed to Malaria in Brazilian Amazon
Source: Vaccines (Basel). 2026 May 8;14(5):423. doi: 10.3390/vaccines14050423 (PMC13211559; doi:10.3390/vaccines14050423)
Supplement: Supplementary file 1 [file vaccines-14-00423-s001.zip › Supplementary Figure S1.pdf]

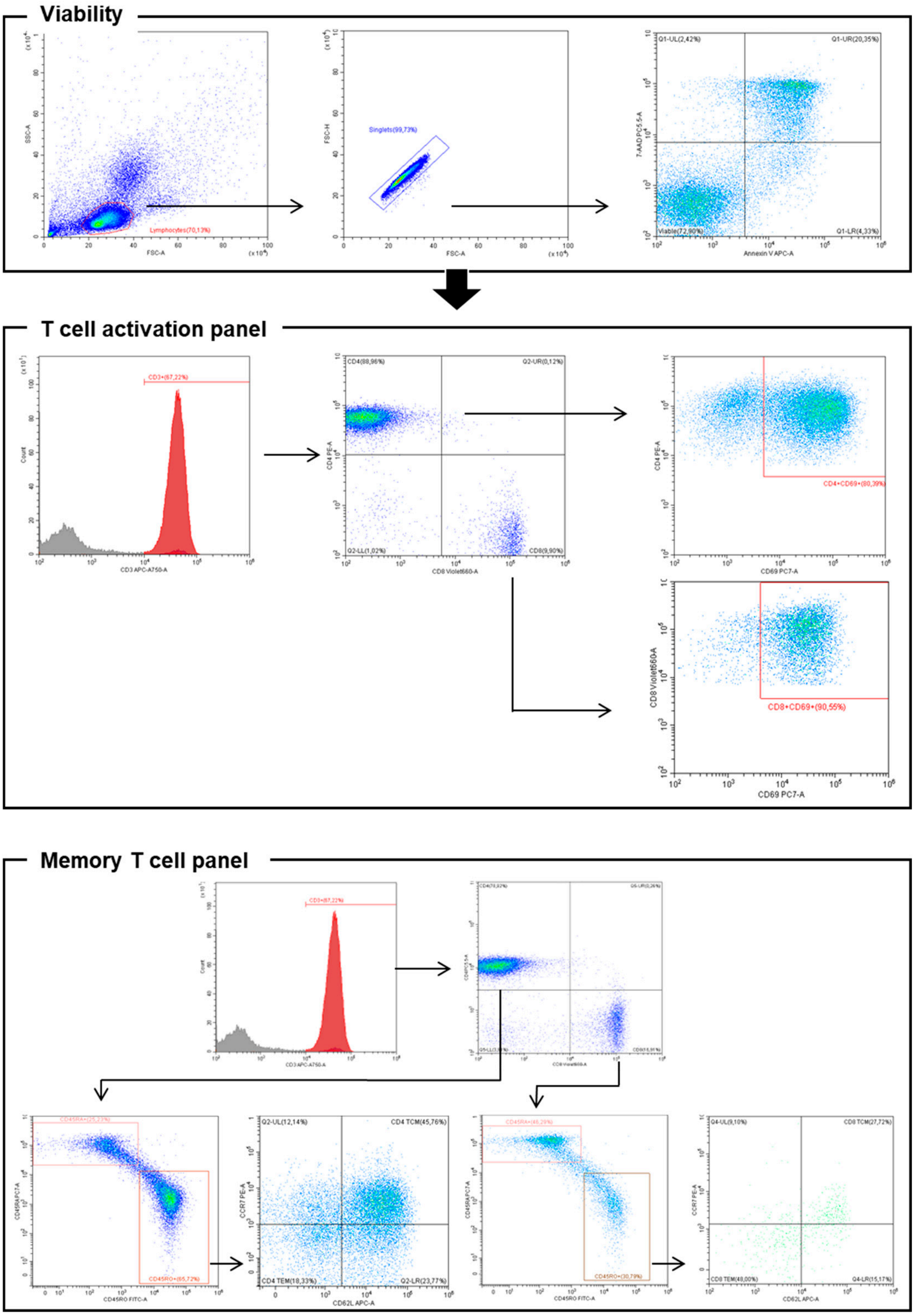

Supplementary Figure S1: Flow cytometry gating strategy for viability, T cell activation, and memory T cell panels. Annexin V/7-AAD staining was used to gate viable lymphocytes (Annexin V$^-$/7-AAD$^-$) for flow cytometry analyses. Flow cytometry analysis of activated CD4 (CD3$^+$CD4$^+$CD69$^+$) and CD8 (CD3$^+$CD8$^+$CD69$^+$) T cells, naive CD4 (CD3$^+$CD4$^+$CD45RA$^+$CD45RO$^-$) and CD8 (CD3$^+$CD8$^+$CD45RA$^+$CD45RO$^-$) T cells, memory CD4 (CD3$^+$CD4$^+$CD45RA$^-$CD45RO$^+$) and CD8 (CD3$^+$CD8$^+$CD45RA$^-$CD45RO$^+$) T cells, T CD4 (CD3$^+$CD4$^+$CD45RA$^-$CD45RO$^+$CD197$^+$CD62L$^+$) and CD8 (CD3$^+$CD8$^+$CD45RA$^-$CD45RO$^+$CD197$^+$CD62L$^+$) central memory cells, and T CD4 (CD3$^+$CD4$^+$CD45RA$^-$CD45RO$^+$CD197$^-$CD62L$^-$) and CD8 (CD3$^+$CD8$^+$CD45RA$^-$CD45RO$^+$CD197$^-$CD62L$^-$) effector memory cells, from one representative experiment.
