# Supplementary material for: Cellular Immune Response and T Cell Epitope Mapping of Plasmodium falciparum Chimeric Vaccine Candidate GMZ2.6c and Its Components (MSP-3, GLURP and Pfs48/45) in Individuals Naturally Exposed to Malaria in Brazilian Amazon
Source: Vaccines (Basel). 2026 May 8;14(5):423. doi: 10.3390/vaccines14050423 (PMC13211559; doi:10.3390/vaccines14050423)
Supplement: Supplementary file 1 [file vaccines-14-00423-s001.zip › Supplementary Figure S2.pdf]

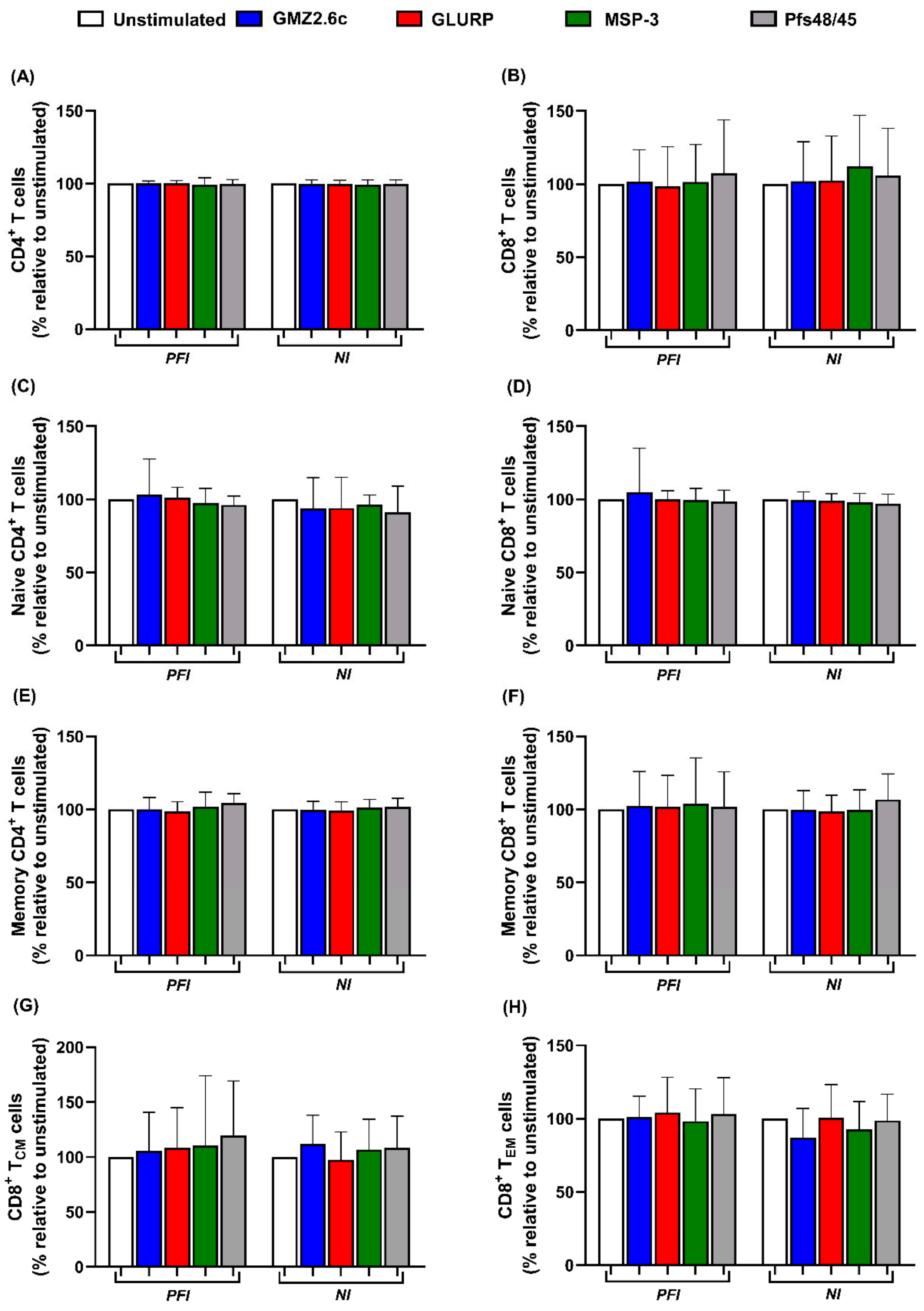


**Supplementary Figure S2:** Lymphocyte subpopulations after GMZ2.6c, GLURP, MSP-3, and Pfs48/45 antigen stimulation. Flow cytometry analysis of total CD4 (**A**) and CD8 (**B**) T cells; naive CD4 (**C**) and CD8 (**D**) T cells; memory CD4 (**E**) and CD8 (**F**) T cells; and CD8 central ($T_{CM}$) (**G**) and effector ($T_{EM}$) (**H**) memory T cells from exposed *P. falciparum*-infected (PFI, n= 20) and non-infected (NI, n= 20) groups after stimulation with GMZ2.6c, GLURP, MSP-3, and Pfs48/45 antigens. Unstimulated PBMCs were used as a baseline to normalize the percentages of stimulated PBMCs (Unstimulated: 100%). Bars represent medians and lines represent interquartile ranges.
