# Supplementary material for: Cellular Immune Response and T Cell Epitope Mapping of Plasmodium falciparum Chimeric Vaccine Candidate GMZ2.6c and Its Components (MSP-3, GLURP and Pfs48/45) in Individuals Naturally Exposed to Malaria in Brazilian Amazon
Source: Vaccines (Basel). 2026 May 8;14(5):423. doi: 10.3390/vaccines14050423 (PMC13211559; doi:10.3390/vaccines14050423)
Supplement: Supplementary file 1 [file vaccines-14-00423-s001.zip › Supplementary Figure S3.pdf]

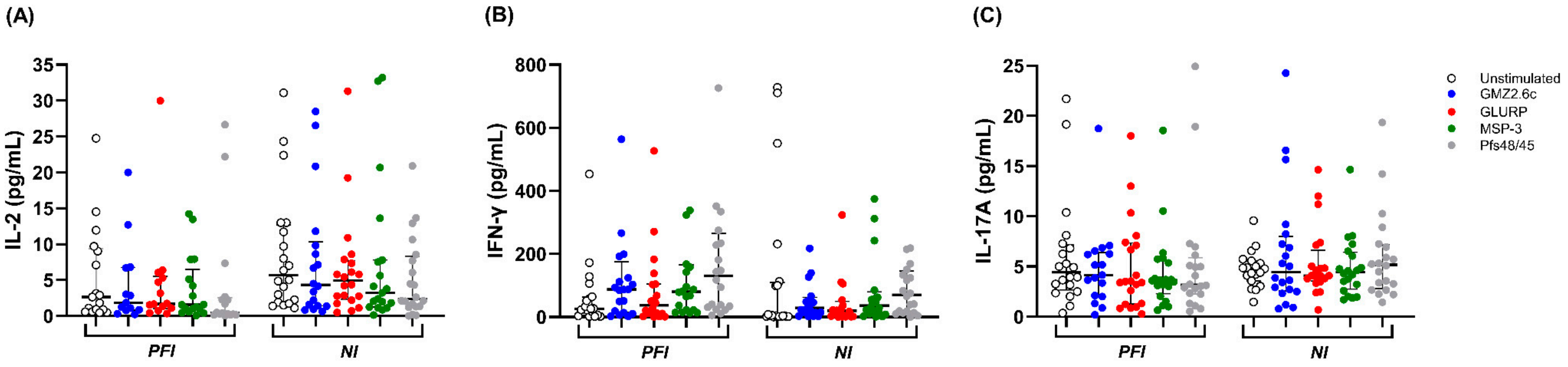

Supplementary Figure S3: PBMC cytokine response under stimulation with GMZ2.6c, GLURP, MSP-3, and Pfs48/45. Concentrations of IL-2 (A), IFN-γ (B), and IL-17A (C) in the supernatant of the PBMC cultures from exposed *P. falciparum*-infected (PFI, n= 20) and non-infected (NI, n= 20) groups. Dots represent individual values and lines represent interquartile range.
