# Supplementary material for: Cellular Immune Response and T Cell Epitope Mapping of Plasmodium falciparum Chimeric Vaccine Candidate GMZ2.6c and Its Components (MSP-3, GLURP and Pfs48/45) in Individuals Naturally Exposed to Malaria in Brazilian Amazon
Source: Vaccines (Basel). 2026 May 8;14(5):423. doi: 10.3390/vaccines14050423 (PMC13211559; doi:10.3390/vaccines14050423)
Supplement: Supplementary file 1 [file vaccines-14-00423-s001.zip › Supplementary Table S1.pdf]

Supplementary Table S1: Flow cytometry staining panels.

Marker	Fluorochrome	Clone	Company	Catalog number
T cell activation panel				
CD3	APC-H7	SK7	BD Pharmingen	560176
CD4	PE	RPA-T4	BD Pharmingen	561844
CD8	BV650	RPA-T8	BD Horizon	563821
CD69	PE-Cy7	L78	BD	335792
Memory T cell panel				
CD3	APC-H7	SK7	BD Pharmingen	560176
CD4	PerCP-Cy5.5	RPA-T4	BD Pharmingen	560650
CD8	BV650	RPA-T8	BD Horizon	563821
CD45RA	PE-Cy7	HI100	BD Pharmingen	560675
CD45RO	BB515	UCHL1	BD Horizon	564529
CD197 (CCR7)	PE	150503	BD Pharmingen	560765
CD62L	APC	DREG-56	BD Pharmingen	559772
